# Hexokinase II Detachment from Mitochondria Triggers Apoptosis through the Permeability Transition Pore Independent of Voltage-Dependent Anion Channels

**DOI:** 10.1371/journal.pone.0001852

**Published:** 2008-03-19

**Authors:** Federica Chiara, Diego Castellaro, Oriano Marin, Valeria Petronilli, William S. Brusilow, Magdalena Juhaszova, Steven J. Sollott, Michael Forte, Paolo Bernardi, Andrea Rasola

**Affiliations:** 1 CNR Institute of Neuroscience and Department of Biomedical Sciences, University of Padova, Padova, Italy; 2 Department of Biological Chemistry and Venetian Institute of Molecular Medicine (VIMM), University of Padova, Padova, Italy; 3 Department of Biochemistry and Molecular Biology, Wayne State University School of Medicine, Detroit, Michigan, United States of America; 4 Laboratory of Cardiovascular Science, Gerontology Research Center, National Institute on Aging, National Institutes of Health, Baltimore, Maryland, United States of America; 5 Vollum Institute, Oregon Health & Science University, Portland, Oregon, United States of America; University of Oldenburg, Germany

## Abstract

Type II hexokinase is overexpressed in most neoplastic cells, and it mainly localizes on the outer mitochondrial membrane. Hexokinase II dissociation from mitochondria triggers apoptosis. The prevailing model postulates that hexokinase II release from its mitochondrial interactor, the voltage-dependent anion channel, prompts outer mitochondrial membrane permeabilization and the ensuing release of apoptogenic proteins, and that these events are inhibited by growth factor signalling. Here we show that a hexokinase II N-terminal peptide selectively detaches hexokinase II from mitochondria and activates apoptosis. These events are abrogated by inhibiting two established permeability transition pore modulators, the adenine nucleotide translocator or cyclophilin D, or in cyclophilin D knock-out cells. Conversely, insulin stimulation or genetic ablation of the voltage-dependent anion channel do not affect cell death induction by the hexokinase II peptide. Therefore, hexokinase II detachment from mitochondria transduces a permeability transition pore opening signal that results in cell death and does not require the voltage-dependent anion channel. These findings have profound implications for our understanding of the pathways of outer mitochondrial membrane permeabilization and their inactivation in tumors.

## Introduction

Hexokinase (HK) initiates all major pathways of intracellular glucose utilization, and type II HK (HK II) couples glycolysis to oxidative phosphorylation by interacting with mitochondria, thus acting as a metabolic sensor [1]. In highly glycolytic, *i.e.* extremely aggressive tumours, mitochondrial HK II activity is increased [2] and fosters cell growth in the hypoxic conditions of neoplastic mass accrual by enhancing glycolysis, which becomes independent of oxygen availability (the Warburg effect, 3). Furthermore, mitochondrial HK II plays an important role in maintaining the integrity of the outer mitochondrial membrane (OMM), thus preventing the release of key apoptogenic molecules from the intermembrane space [2]. Reports indicate that nutrients, via the survival kinase Akt [4], [5], promote HK II binding to the voltage-dependent anion channel [VDAC], [6,7], a protein that allows for the movement of small metabolites across the OMM [8], [9]. Release of HK II transmits a potent death signal [10] that is elicited when GSK3β, a kinase inhibited by Akt, phosphorylates a putative HK docking site on VDAC [11]. However, the role of this pathway remains controversial, as it has been documented that inhibition of GSK-3β results in GSK-3β-mediated resistance to oxidant stress [12].

When associated to HK II, VDAC interacts with antiapoptotic Bcl-2 family members [13]. These might be competed away from VDAC by the pro-apoptotic Bax/Bak proteins after deprivation of growth factors, leading to the formation of a conduit on the OMM fit for the release of apoptogenic proteins [4], [14]. Alternatively, the HK/VDAC interaction could transmit molecular changes to proteins of the inner mitochondrial membrane (IMM), resulting in permeability transition pore (PTP) regulation (reviewed in 2). The PTP is an IMM channel whose opening elicits depolarization, matrix swelling, and consequently cristae unfolding and breaches in the OMM that are pervious to proteins [15], [16].

A distinction between these two possible scenarios would have important consequences both on the comprehension of apoptosis dysregulation in tumors and on the design of therapeutic approaches targeted to selectively eliminate neoplastic cells. Here, we show that cell death mediated by HK detachment from mitochondria is selectively sensitive to modulators of the PTP. Moreover, we demonstrate that VDAC is dispensable for carrying out this process.

## Results

### Clotrimazole detaches HK II from mitochondria but displays non-specific effects

**Figure 1:**
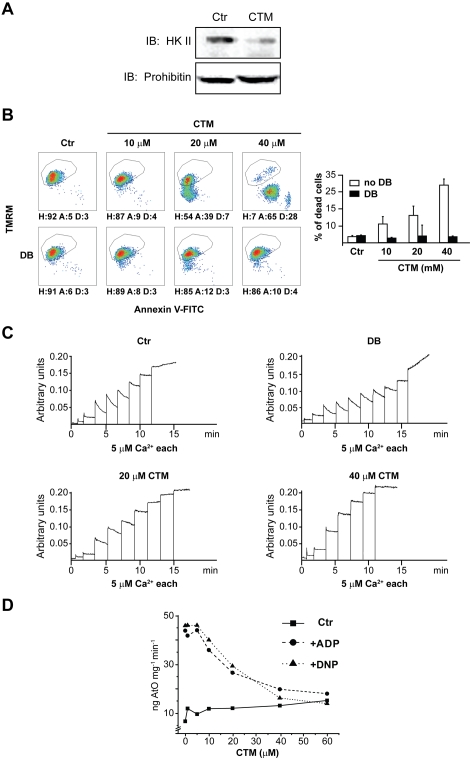
Effects of clotrimazole on cells and mitochondria. (A) HK II detachment from mitochondria following a 1 hour treatment with clotrimazole (CTM, 20 µM) was assessed by Western immunoblot on a mitochondrial fraction of HeLa cells. The blot was also probed with prohibitin as a mitochondrial marker and with actin as a cytosolic marker (negative, not shown), to check for fraction purity. (B) Output of multiparametric FACS analysis show apoptosis induction of HeLa cells exposed for 2 hours to several concentrations of CTM with or without pre-incubation with Debio 025 (DB, 8 µM). Healthy cells (H) are delimited by the quadrant. Apoptotic cells (A) display mitochondrial depolarization (reduced TMRM staining) and/or expose phosphatidylserine on their surface (increased Annexin V-FITC staining). Propidium iodide-positive, *i.e.* dead cells (D), were evaluated on a PI *vs* TMRM diagram (not shown); these cells were excluded from the reported plots and shown as histograms. Numbers are percentages. (C) PTP opening of digitonized HeLa cells measured with the Ca^2+^ retention capacity assay. Calcium Green-5N fluorescence is reported as arbitrary units on the *y* axis. As the probe does not permeate mitochondria, Ca^2+^ uptake into the organelles is displayed by a rapid decrease of the fluorescence spike. Pore inducers or inhibitors reduce or increase, respectively, the threshold Ca^2+^ concentration required to trigger the permeability transition, *i.e.* the number of spikes before a sudden and marked fluorescence increase occurs. Experiments were started by the addition of digitonized cells (not shown) followed by pulses of the indicated concentrations of Ca^2+^. Where indicated, CTM was added to cells 45 minutes before permeabilization with digitonin. Note that CTM reduced the rate of Ca^2+^ uptake into mitochondria. In the upper right plot DB was added before mitochondria to test the PTP-dependence of Ca^2+^ release. (D) Respiration assay performed on mitochondria isolated from mouse liver. The graph shows the rate of oxygen consumption (ng AtO stands for ngatoms of oxygen) *vs.* different concentrations of CTM. The rate of respiration is displayed in basal conditions (▪), after ADP administration (•) or after dinitrophenol administration (▴). All reported results in the Figure are representative of at least four experiments.

We initially induced HK II detachment from the OMM with clotrimazole, a drug that efficiently dissociates HK from mitochondria in several cell models [e.g. 4], [14], [17]. As expected, clotrimazole detached HK II from mitochondria of HeLa cells (Figure 1A) and of human T lymphoma Jurkat cells (data not shown). Consistent with published reports, this treatment prompted a marked and concentration-dependent cell death (Figure 1B, upper row).

We asked whether HK II detachment from the OMM could induce cell death by opening the mitochondrial PTP. The molecular composition of the PTP is at present unsolved. Nonetheless, pharmacological and genetic evidences identify cyclophilin (CyP-D), a matrix peptidyl-prolyl *cis-trans* isomerase, and the adenine nucleotide translocator (ANT), a carrier that exchanges adenine nucleotides across the IMM, as pore regulators [18]–[22]. Therefore, cells were pretreated with the cyclophilin inhibitor MeAla3EtVal4-cyclosporin (Debio 025), selectively targeting CyP-D in mitochondria, and thereby desensitizing the mitochondrial PTP without inhibiting calcineurin [23], [24]. Remarkably, cell death was fully abrogated (Figure 1B, lower row). We then directly assessed occurrence of PTP opening by measuring the threshold Ca^2+^ required to open the PTP with the sensitive Ca^2+^ retention capacity (CRC) test. Permeabilized whole-cells were triggered with trains of Ca^2+^ pulses in the presence of a Ca^2+^-sensitive fluorescent probe that does not permeate mitochondria (see Methods). Ca^2+^ uptake into mitochondria was therefore displayed as a rapid fluorescence drop. When the PTP opened, the ensuing Ca^2+^ release from mitochondria was assessed as a sudden fluorescence increase. We found that clotrimazole inhibited Ca^2+^ uptake into mitochondria, *i.e.* the slope of the fluorescence reduction after each pulse (Figure 1C), and this same effect was also observed in isolated mitochondria (not shown). We also found that clotrimazole dramatically decreased ADP- and uncoupler-stimulated respiration in a concentration-dependent manner (Figure 1D); similar results were obtained if succinate (in the presence of rotenone) rather than glutamate/malate was used as a substrate (not shown). Note that respiration and mitochondrial CRC experiments were performed in organelles from liver, and hepatocytes have virtually no HK II [2]. Thus, clotrimazole inhibited respiration by binding to a molecular target other than HK II, and it appears to be toxic to mitochondria independently of its activity on HK II and/or PTP. As dissecting the pharmacology of clotrimazole was beyond the scope of this study, we achieved HK II detachment from mitochondria with a different strategy.

### A HK II peptide specifically detaches HK II from mitochondria and induces cell death

**Figure 2:**
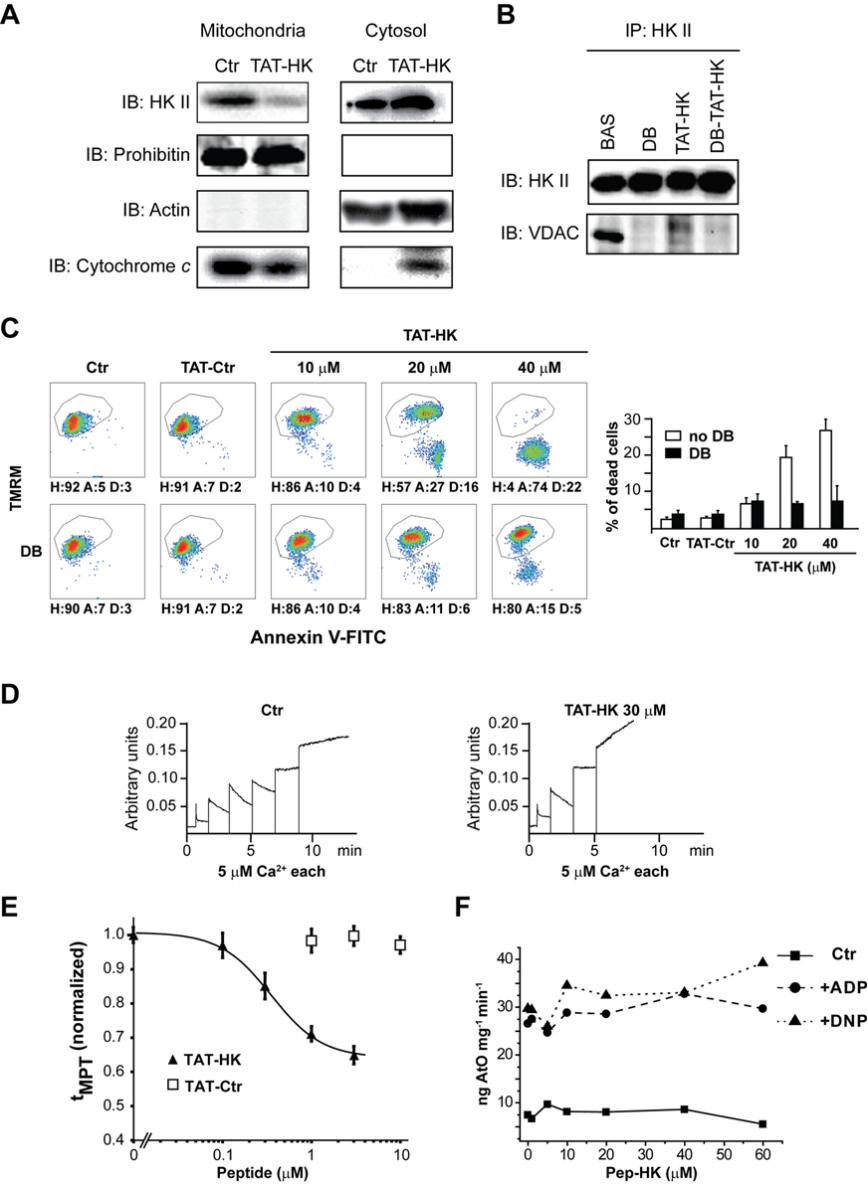
Selective detachment of HK II from mitochondria induces PTP opening and cell death. (A) HK II detachment from mitochondria of HeLa cells following a 1-hour treatment with TAT-HK (20 µM). Western immunoblots of a mitochondrial and of a cytosolic fraction show a redistribution of both HK II and cytochrome *c* into the cytosol. Blots were also probed with prohibitin as a mitochondrial marker and with actin as a cytosolic marker to check for fraction purity. (B) HK II immunoprecipitation in HeLa cells kept in control conditions or treated for 1 hour with Debio 025 (DB, 8 µM), TAT-HK (20 µM), or both. Co-immunoprecipitation of VDAC is shown. (C) Output of multiparametric FACS analyses show apoptosis induction of HeLa cells exposed for 2 hours to a control peptide linked to the TAT sequence (TAT-Ctr, 40 µM) or to the reported concentrations of TAT-HK with or without pre-incubation with Debio 025 (DB, 8 µM). Diagrams and percentages of the different cell populations are as in Fig. 1B. (D) PTP opening of digitonized HeLa cells measured with the Ca^2+^ retention capacity assay. Experiments were started by the addition of cells (not shown) followed by pulses of the indicated concentrations of Ca^2+^; where indicated, TAT-HK (30 µM) was added to cells 45 minutes before permeabilization with digitonin. TAT-Ctr (40 µM) did not change the number of Ca^2+^ pulses (not shown). Calcium Green-5N fluorescence is reported as arbitrary units on the *y* axis. (E) MPT susceptibility to ROS (t_MPT_) in intact cardiac myocytes is significantly enhanced by TAT-HK while the negative control peptide TAT-Ctr has no effect. t_MPT_ measurements were performed after 1 hr treatment with peptides. Figure represents four independent experiments with 50–60 cells per groups examined. (F) Respiration assay performed on mouse liver mitochondria. The rate of oxygen consumption (ng AtO stands for ngatoms of oxygen) is plotted *vs.* different concentrations of the HK peptide. The rate of respiration is displayed in basal conditions (▪), after ADP administration (•) or after dinitrophenol administration (▴). All reported measures in the Figure are representative of at least four experiments.

Peptides corresponding to the N-terminal hydrophobic domain of HK were shown to effectively displace it from VDAC [4], [14], suggesting that the latter is required for HK binding to mitochondria [2], [25], [26]. We reasoned that this approach could avoid the unspecific toxic effects observed with clotrimazole. The commercially available fusion protein Antennapedia-HK II [14] was not suitable for our experiments since it has been recently demonstrated that the internalization domain of the Antennapedia homeoprotein itself exhibits confounding effects on the mitochondrial permeability transition ROS threshold in cardiac myocytes [27]. We therefore linked the N-terminal 15 amino acids of HK II to a HIV-1 TAT sequence to allow its entry into the cell. Treatment of HeLa cells with this oligopeptide, called TAT-HK, efficiently displaced HK II from mitochondria (Figure 2A), and induced cytochrome *c* (cyt *c*) release into the cytosol (Figure 2A), a hallmark of mitochondrial apoptosis. TAT-HK also disrupted the interaction between HK II and its reported binding partner on the OMM, VDAC (Figure 2B). However, in keeping with a previous work in which cyclosporin A was used [17], we found that the HK II/VDAC binding could also be abrogated by Debio 025, irrespective of the presence of the TAT-HK peptide (Figure 2B). We excluded the possibility that this effect of Debio 025 was due to inhibition of cytosolic CyP-A, as this protein was not detectable in our mitochondrial preparations (not shown). TAT-HK caused a concentration-dependent apoptosis, whereas a peptide obtained by linking an unrelated sequence to the same TAT motif was totally ineffective (Figure 2C, upper row). Debio 025 abrogated TAT-HK-induced apoptosis (Figure 2C, lower row), indicating that this apoptosis was dependent on PTP opening. Moreover, this treatment elicited PTP opening in permeabilized HeLa cells, as assessed by the reduction of the number of Ca^2+^ pulses in CRC assays, without affecting the rate of Ca^2+^ uptake into mitochondria (Figure 2D). To extend these results, the activity of the PTP was assessed in a model of excitable cells with high metabolic demand expressing mostly HK II, *i.e.* freshly isolated adult rat cardiac myocytes [28]. We previously developed a method that allows precise determination of the PTP sensitivity to oxidant stress in intact cardiac myocytes [29]. These cells turned out to be extremely sensitive to the toxic effect of the peptide. TAT-HK showed a concentration-dependent effect on the ROS threshold for mitochondrial permeability transition (MPT) induction (*t*_MPT_). *t*_MPT_ was significantly decreased in myocytes exposed to >0.1 µM TAT-HK for 1 hr, and 10 µM TAT-HK induced fast collapse of the mitochondrial membrane potential followed by cell death (Figure 2E and data not shown). The control peptide had no effect over the concentration range tested (up to10 µM).

Unlike clotrimazole, the HK peptide did not affect mitochondrial respiration, as measured on isolated mitochondria (Figure 2F).

### The PTP modulators CyP-D and ANT are involved in TAT-HK-elicited apoptosis

**Figure 3:**
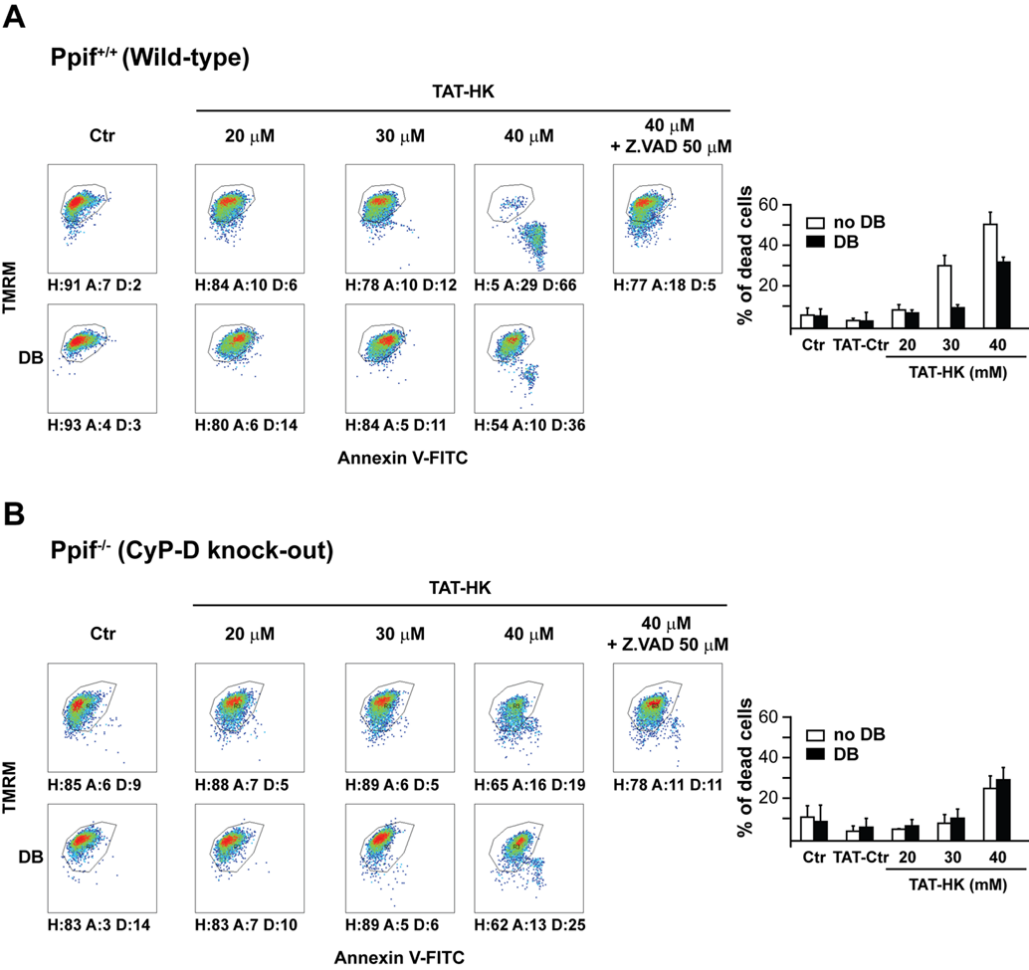
CyP-D modulates apoptosis triggered by detachment of HK II from mitochondria. (A, B) Output of multiparametric FACS analyses show apoptosis induction in fibroblasts obtained either from wild-type mice (A) or from mice in which the *Ppif* gene encoding for CyP-D was ablated (B). Cells were exposed for 2 hours to the reported concentrations of TAT-HK with or without pre-incubation with Debio 025 (DB, 8 µM) or with a pan-caspase inhibitor (Z.VAD-fmk, 50 µM). Diagrams and percentages of the different cell populations are indicated as in Fig. 1B. All reported measures in the Figure are representative of at least four experiments.

**Figure 4:**
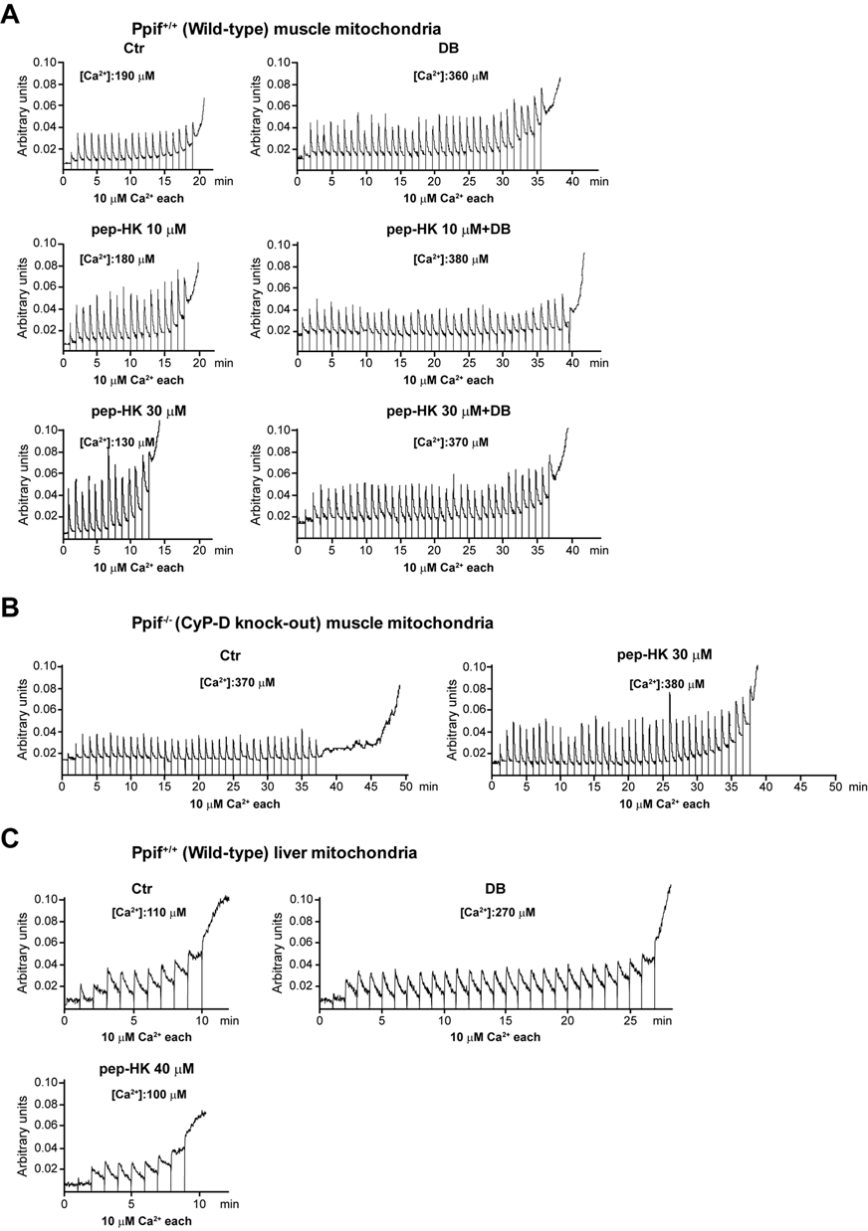
CyP-D modulates PTP opening triggered by detachment of HK II from mitochondria. CRC experiments were performed on mitochondria isolated from muscle of either wild-type (A) or CyP-D knock-out mice (B), or from liver of wild-type animals (C). Assays were started by the addition of mitochondria followed by pulses of the indicated concentrations of Ca^2+^; where indicated, the reported concentrations of the HK peptide lacking the TAT sequence and/or Debio 025 (DB, 8 µM) were added to mitochondria 5 minutes before recordings. Calcium Green-5N fluorescence is reported as arbitrary units on the *y* axis. Total Ca^2+^ loading required to open the pore is reported for each condition, and all reported results are representative of at least four experiments. Treatment of mitochondria with the control peptide (40 µM) or with a HK peptide harbouring the TAT sequence did not change the number of Ca^2+^ pulses (not shown). All reported measures in the Figure are representative of at least four experiments.

In order to further characterize the molecular events determined by HK displacement from the OMM, we investigated the role of CyP-D, the mitochondrial target of Debio 025. We carried out these studies in immortalized fibroblasts from either wild-type or CyP-D knock-out mice [19] which both express HK II (data not shown). TAT-HK induced cell death in wild-type fibroblasts, and this was fully inhibited either by Debio 025 or by the pan-caspase inhibitor Z.VAD-fmk (Figure 3A). Notably, the peptide was only a weak apoptosis inducer in cells derived from CyP-D knock-out mice, and its limited effect was insensitive to Debio 025 but still inhibitable by Z.VAD-fmk (Figure 3B). The unrelated TAT-linked peptide was again ineffective (data not shown). The mandatory role of CyP-D in apoptosis triggered by HK II detachment was confirmed by direct assessment of pore regulation. In fact, mitochondria obtained from wild-type muscles, which express high levels of HK II bound onto their surface, were highly sensitive to pore opening induced by the peptide in a Debio 025-inhibitable fashion (Figure 4A), whereas PTP opening was not susceptible to the HK II peptide in organelles originated from CyP-D knock-out muscles (Figure 4B). Furthermore, the peptide was completely unable to sensitize the pore in wild-type mitochondria obtained from liver (Figure 4C), in which the HK II level is negligible. These observations consistently indicate that CyP-D plays an important role in PTP opening and apoptosis induction due to selective HK II detachment from mitochondria.

**Figure 5:**
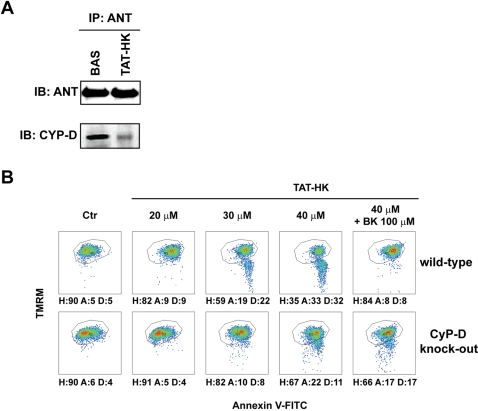
ANT modulates apoptosis triggered by detachment of HK II from mitochondria. (A) Western blot of ANT immunoprecipitation. Co-immunoprecipitation of CyP-D in wild-type mouse fibroblasts is shown either in control conditions or after a 1 hour treatment with TAT-HK. (B) Output of multiparametric FACS analyses show apoptosis induction in fibroblasts obtained either from wild-type mice (upper row) or from CyP-D knock-out animals (lower row). Cells were exposed for 2 hours to the stated concentrations of TAT-HK with or without a 3 hour pre-incubation with bongkrekate (BK, 100 µM). Diagrams and percentages of the different cell populations are indicated as in Fig. 1B. All reported measures in the Figure are representative of at least four experiments.

We then asked whether displacement of HK II from mitochondria elicited apoptosis by affecting other pore regulators. Pharmacological ligands of the IMM protein ANT modulate PTP opening, but this effect is lost in mitochondria obtained from ANT knock-out cells. As these mitochondria still undergo the permeability transition [22], ANT is dispensable as a pore component, but it operates as a PTP regulator. In this framework, we reasoned that ANT could funnel a conformational change from HK II on the mitochondrial surface to modulate the pore in the inner membrane. In keeping with the presence of a signal transduced by HK II displacement to pore regulators, we observed through immunoprecipitation experiments that the expected [30] interaction between ANT and CyP-D was disrupted by the TAT-HK peptide (Figure 5A). Remarkably bongkrekate, an ANT ligand that stabilizes it in the “m” conformation and acts as an inhibitor of the PTP [31], abrogated cell death triggered by TAT-HK in wild-type but not in CyP-D-knock-out cells (Figure 5B). The lethal effect of TAT-HK could not be rescued by the F_O_F_1_ ATP synthase inhibitor oligomycin, suggesting that the effect of bongkrekate is not secondary to inhibition of ATP synthesis (data not shown).

### VDAC is dispensable for TAT-HK-elicited apoptosis

**Figure 6:**
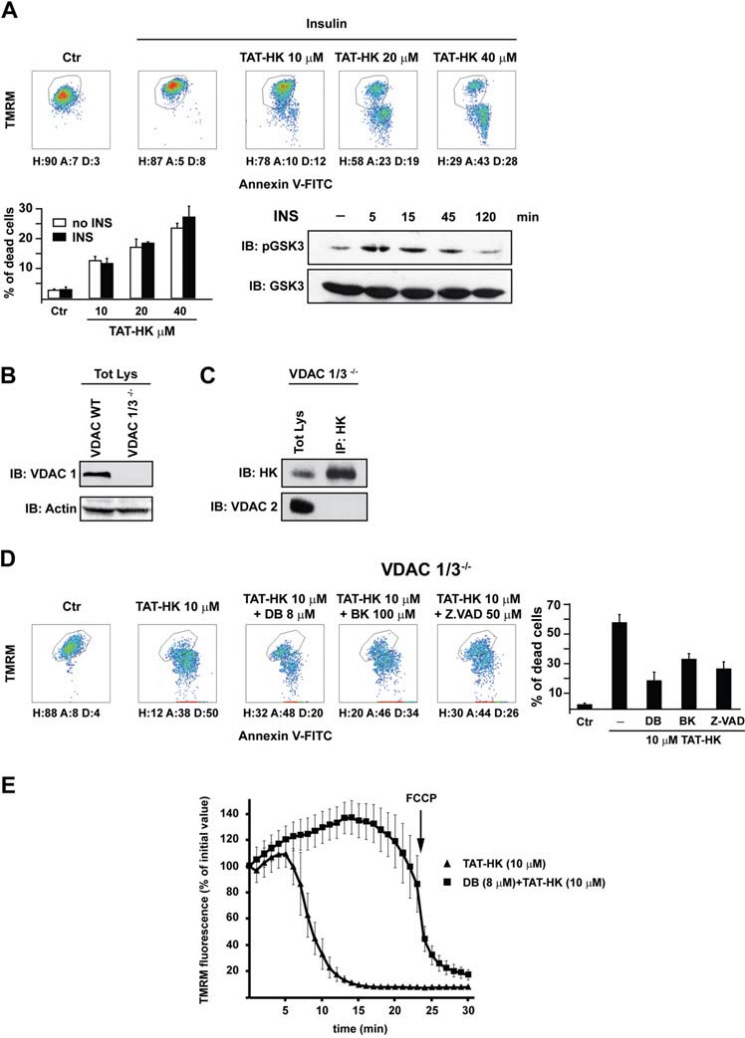
Neither insulin nor VDAC modulate apoptosis triggered by detachment of HK II from mitochondria. (A) After 10 minutes of insulin (5 µg/ml) stimulation, HeLa cells were exposed for 2 hours to the reported concentrations of TAT-HK and apoptosis assayed as in Figure 1B. Western immunoblot shows GSK3β inhibition (*i.e.* Ser9 phosphorylation) by insulin. (B) HK II and VDAC expression in MEF obtained from either wild-type or VDAC 1/3 knock-out mice were assessed by Western immunoblot. VDAC expression was checked with an anti-VDAC1antibody, protein loading with an anti-actin antibody. (C) Immunoprecipitation of HK II and rehybridization with an anti VDAC2 in VDAC1/3 knock-out MEFs. (D) Output of multiparametric FACS analyses show apoptosis induction in VDAC1/3 knock-out MEFs. Cells were exposed for 1 hour to the stated concentrations of TAT-HK with or without pre-incubation with Debio 025 (DB, 8 µM), bongkrekate (BK, 100 µM) or Z.VAD-fmk (50 µM). Diagrams and percentages of the different cell populations are indicated as in Fig. 1B. (E) Mitochondrial depolarization after cell treatment with TAT-HK (10 µM) with or without pre-incubation with Debio 025 (8 µM), as assessed by decreased TMRM staining by epifluorescence microscopy. All reported measurements are representative of at least four experiments.

Following the current model, VDAC phosphorylation by GSK3β prompts HK II detachment from VDAC and induces apoptosis. This is inhibited by growth factors through Akt-dependent inactivation of GSK3β. We therefore stimulated HeLa cells with insulin, a powerful Akt inducer, to test whether this could inhibit peptide-mediated apoptosis. Surprisingly, we found that insulin did not affect TAT-HK elicited apoptosis (Figure 6A).

To formally clarify whether VDAC is essential for the PTP-opening signal conveyed by HK II displacement, we exploited cells obtained from VDAC knock-out animals. Three distinct VDAC isoforms exist in mammals [9]. We utilized mouse embryo fibroblasts (MEF) obtained from VDAC1/3 knock-out animals (Figure 6B), as triple knock-out mice are not viable [32]. According to reports indicating that HK binding is a specific feature of the VDAC1 isozyme [33], we could not detect any co-immunoprecipitation between the residual VDAC2 isoform and HK II in these cells (Figure 6C). We next addressed whether VDAC1/3^−/−^ cells were protected from the noxious activity of the TAT-HK peptide. To our surprise, VDAC1/3^−/−^ MEFs were highly sensitive to the peptide (Figure 6D) in a concentration-dependent manner (data not shown). We also found that cell death was inhibited by Debio 025, by the pan-caspase inhibitor Z.VAD-fmk and by bongkrekate (Figure 6D). These observations support a model in which HK II displacement from mitochondria triggers a PTP-dependent but VDAC-independent apoptotic cascade. This model is strongly supported by direct measurements of pore opening in VDAC1/3^−/−^ MEFs treated with TAT-HK, where the peptide induced a fast mitochondrial depolarization that was abrogated by Debio 025 (Figure 6E).

## Discussion

Mitochondrial HK II integrates information from growth factor signalling, and acts as a powerful anti-apoptotic gatekeeper to suppress cell death in conditions of adequate nutrition. This is of pivotal importance in cancer, because neoplastic cells display an aberrant hyper-activation of growth factor-induced metabolic pathways; the related overexpression and activation of HK II on mitochondria confers a competitive edge to the most aggressive cancers by increasing their glycolytic metabolism [3], and thus supporting cell proliferation when the tumour mass outgrows the surrounding blood vessels [2]. It is therefore crucial for anti-neoplastic strategies to dissect in detail the mechanisms by which HK II controls the death/survival rheostat of cells.

In this report, we have confirmed that HK II dissociation from mitochondria is a strong pro-apoptotic stimulus, and established that this form of apoptosis requires opening of the mitochondrial PTP independently of VDAC. This observation has conceptual and therapeutical implications. Loss of mitochondrial membrane integrity constitutes a point of no-return in cell commitment to death. Critical effector mechanisms include loss of ATP production and the eventual release from the mitochondrial intermembrane space of proteins with key functions in cell dismantling. The size of these proteins entails specific forms of OMM permeabilization for their release, whose molecular mechanisms remain controversial. One possibility is the direct formation of large channels on the OMM, composed either by proapoptotic Bcl-2 family proteins, or by association of these proteins with mitochondrial membrane channels; proteins might also be funnelled through lipidic pores. Alternatively, opening of the PTP in the IMM could indirectly induce ruptures in the OMM that are large enough to allow release of apoptogenic protein (reviewed in 16,34). The two mechanisms are not mutually exclusive. For instance, cristae remodelling could be required as a PTP-dependent first step to trigger channel opening on the OMM [35]; and differences in the mode of OMM permeabilization could depend on the cell type, the stress stimulus and the energetic conditions of the cell.

Our data indicate that HK release from the OMM prompts a death stimulus that requires PTP opening. The detailed mechanism cannot be defined at present, as the molecular composition of the PTP remains an unsolved riddle. In fact, all suspected pore components, *i.e.* CyP-D, VDAC and ANT, underwent a rigorous analysis based on the ablation of their coding genes followed by investigation of PTP properties, and none of them turned out to be part of the pore, even if ANT and CyP-D are PTP regulators [18]–[22], [32], [36]. Here, we found that both ANT and CyP-D are key regulators of apoptosis induced by mitochondrial HK II displacement. Treatment with the TAT-HK peptide disrupts the interaction between ANT and CyP-D, suggesting that HK II partitioning away from mitochondria propagates a conformational change directed from molecules associated to the OMM towards structures of the inner membrane, where the PTP resides [15]. This might trigger PTP opening, resulting in the release of apoptogenic proteins and in cell death.

Our results challenge the view that VDAC is the mitochondrial determinant of cell survival or death, acting as a hub that binds either anti- or pro-apoptotic Bcl-2 family members depending on the presence or absence of HK II, respectively [2]. This model did not explain how HK dissociation from mitochondria could induce OMM permeabilization even in the absence of Bax and Bak [4]. Here we demonstrate that HK II/VDAC binding is also disrupted in conditions that do not promote cell death, *i.e.* following treatment with Debio 025. More importantly, in cells lacking both VDAC1 and VDAC3, no binding between HK II and the residual VDAC2 could be detected, yet displacement of HK II from mitochondria prompted massive cell death. In addition, the interaction between VDAC and HK II was reported to be inhibited by GSK3β-mediated VDAC phosphorylation, and stimulated by Akt-dependent inactivation of GSK3β [11], and this would protect from cell death. However, we found that treating cells with insulin, a powerful Akt activator, cells were not protected from apoptosis elicited by mitochondrial HK II displacement. Again, these results argue against a mandatory cell survival role of a transduction pathway that should convey signals to VDAC. Thus, VDAC appears to be dispensable for cell death elicited by HK II detachment from mitochondria, while resistance to cell death in highly glycolytic tumors appears to be critically dependent on preventing opening of the mitochondrial PTP.

## Materials and Methods

### Chemicals, antibodies and cells

FITC-conjugated Annexin-V was from Boehringer Mannheim (Indianapolis, IN); Calcium Green-5N and tetramethylrhodamine methyl ester (TMRM) were from Molecular Probes (Eugene, OR); the caspase inhibitor Z.VAD-fluoro-methyl-ketone (Z.VAD-fmk) was from Bachem (Subendorf, Switzerland); all other chemicals were from Sigma (St. Louis, MO). Debio 025 was a generous gift of Debiopharm (Lausanne, Switzerland). The mouse monoclonal anti-prohibitin antibody was from Lab Vision (Fremont, CA); the mouse monoclonal anti-actin antibody was from Sigma; the monoclonal anti-CyP-D antibody was from Calbiochem (San Diego, CA); the rabbit polyclonal anti VDAC1 antibody was from Abcam, (Cambridge, UK); the goat polyclonal anti HK II, anti VDAC2 and anti ANT antibodies and the mouse monoclonal anti GSK-3 antibody were from Santa Cruz Biotechnology (Santa Cruz, CA); the mouse monoclonal anti-cytochrome *c* antibody was from Becton Dickinson Pharmingen (Franklin Lakes, NJ); the rabbit monoclonal anti Bcl-X_L_ and anti phospho Ser-GSK-3 antibodies were from Cell Signaling (Beverly, MA). Diaphragm adult fibroblasts were obtained by SV40 immortalization of primary cells from wild-type and *Ppif^−/−^* mice; mouse embryo fibroblasts (MEF) from wild-type and *VDAC1/3^−/−^* mice were obtained as described [37]. Apoptosis inducers were added to exponentially growing cells in the absence of serum. Chemical inhibitors of caspases, CyP-D or ANT were added three hours before starting apoptosis induction. As in the following, each experiment was repeated at least four times.

### Flow cytometry analysis of apoptosis induction

Flow cytometry recordings of apoptotic changes were performed as described [38], [39]. Briefly, after induction of apoptosis, cells were resuspended in 135 mM NaCl, 10 mM HEPES, 5 mM CaCl_2_ and incubated for 15 min at 37°C in FITC-conjugated Annexin-V, TMRM (200 nM) and propidium iodide (PI, 1 µg/ml), to detect mitochondrial depolarization (reduced TMRM staining), phosphatidylserine exposure on the cell surface (increased FITC-conjugated Annexin-V staining), and loss of plasma membrane integrity (PI permeability and staining). Samples were analyzed on a FACSCalibur flow cytometer (Becton Dickinson, San Diego, CA, USA). Data acquisition was performed using CellQuest software and data analysis with WinMDI software. Each experiment was repeated at least four times, and consistency of data allowed to show one representative experiment for each condition.

### Isolation of mouse mitochondria

Mitochondria were isolated from livers and muscles of wild-type and *Ppif^−/−^* C57BL/6 mice [19] through sequential centrifugations, as described [40]. All procedures were carried out at 0–4°C.

### Measurement of mitochondrial Ca^2+^ retention capacity

The Ca^2+^ retention capacity (CRC) assay was used to assess PTP opening following trains of Ca^2+^ pulses [40] and measured fluorimetrically at 25°C in the presence of the Ca^2+^ indicator Calcium Green-5N (1 µM; λ_exc_: 505 nm; λ_em_: 535 nm; Molecular Probes). We performed CRC experiments either on isolated mitochondria or on whole cells placed in an isotonic buffer (130 mM KCl, 1 mM Pi-Tris, 10 mM Tris/MOPS, 1 mM EGTA/Tris, pH 7.4) in the presence of 2 µM rotenone/5 mM succinate or 250 mM glutamate/125 mM malate and of 10 µM cytochrome *c*. Whole cell CRC was carried out after plasma membrane permeabilization with the non-ionic detergent digitonin, which is highly selective for cholesterol-enriched membranes and does not damage mitochondrial membranes. Cells were washed twice in a buffer composed of 130 mM KCl, 10 mM Tris/MOPS, 1 mM Pi-Tris, 1 mM EGTA/Tris, pH 7.4, and then incubated with 50–150 µM digitonin (depending on the cell type) for 15 min at 4°C. Digitonin was then washed away by spinning cells twice in a cytosolic-like solution (130 mM KCl, 10 mM Tris/MOPS, 1 mM Pi-Tris, 0.1 mM EGTA/Tris, pH 7.4), and the number of cells carefully assessed before starting each experiment.

### Mitochondrial respiration assay

Mitochondrial oxygen consumption was measured polarographically at 25°C with a Clark oxygen electrode (Yellow Springs Instruments, OH, USA). Mitochondria were incubated in a solution of 130 mM KCl, 10 mM Tris-Mops, 1 mM Pi-Tris, 20 µM EGTA-Tris and 250 mM glutamate/125 mM malate or 2 µM rotenone/5 mM succinate to assay basal respiration (state 4). ADP (200 µM) was subsequently added to measure state 3 respiration, followed by the uncoupling agent dinitrophenol (DNP, 100 µM) to assess the maximal respiration rate.

### Cell lysis, fractionation and Western immunoblot analysis

Total cell extracts were prepared at 4°C in 135 mM NaCl, 20 mM Tris-HCl pH 7.5, 1 mM CaCl_2_, 1% NP40 or 1% Triton X-100 in the presence of phosphatase and protease inhibitors. To prepare mitochondrial extracts, cells were placed cells in a hypotonic solution (10 mM Tris-HCl pH 7.6, 10 mM KCl, 150 µM MgCl_2_) and homogenized at 4°C. Mitochondria were then isolated by differential centrifugation. For immunoprecipitations, 1000–1500 µg of proteins per reaction were incubated with antibodies conjugated to protein A- or protein G-Sepharose beads (Pharmacia, Pfizer, Cambridge, MA) at 4°C overnight. Negative controls were performed by incubating lysates on conjugated beads in the absence of primary antibodies. Samples were then separated in reducing conditions on SDS-polyacrylamide gels and transferred onto Hybond-C Extra membranes (Amersham, Little Chalfont, UK). Primary antibodies were incubated 16 hours at 4°C, and horseradish peroxidase-conjugated secondary antibodies were added for 1 hour. Proteins were visualized by enhanced chemiluminescence (Amersham).

### Peptide synthesis

Peptides MIASHLLAYFFTELNβA-GYGRKKRRQRRRG (TAT-HK) and GYGRKKRRQRRRG-βA-EEEAKNAAAKLAVEILNKEKK (TAT-Ctr) were synthesized by a solid phase method using an automatized peptide synthesizer (model 431-A, Applied Biosystems, Foster City, CA). The fluoren-9-ylmethoxycarbonyl (Fmoc) strategy [41] was used throughout the peptide chain assembly, utilizing 2-(1H-benzotriazol-1-yl)-1,1,3,3-tetramethyluronium hexafluorophosphate (HBTU) and 1-hydroxybenzotriazole (HOBt) as coupling reagents. HMPA PEGA resin (Novabiochem, Bad Soden, Germany) was used as solid support. The side-chain protected amino acid building blocks used were: Fmoc-Tyr(tert-butyl), Fmoc-Glu(tert-butyl), Fmoc-Ser(tert-butyl), Fmoc-Thr(tert-butyl), Fmoc-Lys(tert-butyloxycarbonyl), Fmoc-His(trityl), Fmoc-Asn(trityl), Fmoc-Gln(trityl), and Fmoc-Arg(2,2,4,6,7-pentamethyldihydrobenzofuran-5-sulfonyl). Cleavage of the peptides was performed by reacting the peptidyl-resins with a mixture containing TFA/H20/thioanisole/ethanedithiol/phenol (10 ml/0.5 ml/0.5 ml/0.25 ml/750 mg) for 2.5 h. Crude peptides were purified by a preparative reverse phase HPLC. Molecular masses of the peptides were confirmed by mass spectroscopy with direct infusion on a Micromass ZMD-4000 Mass Spectrometer (Waters-Micromass). The purity of the peptides was about 95% as evaluated by analytical reverse phase HPLC.

### Mitochondrial membrane potential assay

This was measured in the presence of 20 nM TMRM as described elsewhere [24], [42]. Fluorescent images were acquired with an Olympus (Center Valley, PA) IX71/IX51 inverted microscope equipped with a xenon light source and with a 12-bit digital cooled charge-coupled device (CCD) camera (Micromax, Princeton Instruments, Trenton, NJ). For detection of fluorescence, 568 ± 25-nm bandpass excitation and 585-nm longpass emission filter settings were used. Images were collected with an exposure time of 100 msec. Data were acquired and analyzed by using Cell^R^ software (Olympus). Clusters of 10–30 mitochondria were acquired every 2 min, and fields not containing cells were taken as the background.

### Determination of mitochondrial permeability transition (MPT) ROS threshold (*t*_MPT_)

Cardiac myocyte isolation, confocal microscopy and determination of MPT ROS threshold have been described [12], [29]. Briefly, isolated myocytes loaded with 125 nM TMRM were exposed *in vitro* to conditions that mimic oxidative stress by repetitive laser line-scanning of a row of ∼25 mitochondria at 2 Hz with an LSM-510 confocal microscope (Carl Zeiss Inc., Jena, Germany) with excitation at 543nm and collecting emission at >560 nm using a 63x/1.4 oil immersion objective. This results in controlled, incremental photodynamic production of ROS in the scanned area with consequent MPT induction. MetaMorph image analysis software (Molecular Devices Corp., Downingtown PA) was used to calculate *t*_MPT_ and was defined as average time required to induce MPT in targeted mitochondria.

## References

[1] Wilson JE (2003). Isozymes of mammalian hexokinase: structure, subcellular localization and metabolic function.. J Exp Biol.

[2] Mathupala SP, Ko YH, Pedersen PL (2006). Hexokinase II: cancer's double-edged sword acting as both facilitator and gatekeeper of malignancy when bound to mitochondria.. Oncogene.

[3] Warburg O (1956). On the origin of cancer cells.. Science.

[4] Majewski N, Nogueira V, Bhaskar P, Coy PE, Skeen JE (2004). Hexokinase-mitochondria interaction mediated by Akt is required to inhibit apoptosis in the presence or absence of Bax and Bak.. Mol Cell.

[5] Rathmell JC, Fox CJ, Plas DR, Hammerman PS, Cinalli RM (2003). Akt-directed glucose metabolism can prevent Bax conformation change and promote growth factor-independent survival.. Mol Cell Biol.

[6] Pastorino JG, Hoek JB (2003). Hexokinase II: the integration of energy metabolism and control of apoptosis.. Curr Med Chem.

[7] Vyssokikh M, Brdiczka D (2004). VDAC and peripheral channelling complexes in health and disease.. Mol Cell Biochem.

[8] Colombini M (2004). VDAC: the channel at the interface between mitochondria and the cytosol.. Mol Cell Biochem.

[9] Shoshan-Barmatz V, Israelson A, Brdiczka D, Sheu SS (2006). The voltage-dependent anion channel (VDAC): function in intracellular signalling, cell life and cell death.. Curr Pharm Des.

[10] Zaid H, Abu-Hamad S, Israelson A, Nathan I, Shoshan-Barmatz V (2005). The voltage-dependent anion channel-1 modulates apoptotic cell death.. Cell Death Differ.

[11] Pastorino JG, Hoek JB, Shulga N (2005). Activation of glycogen synthase kinase 3beta disrupts the binding of hexokinase II to mitochondria by phosphorylating voltage-dependent anion channel and potentiates chemotherapy-induced cytotoxicity.. Cancer Res.

[12] Juhaszova M, Zorov DB, Kim SH, Pepe S, Fu Q (2004). Glycogen synthase kinase-3beta mediates convergence of protection signaling to inhibit the mitochondrial permeability transition pore.. J Clin Invest.

[13] Zhou H, Hou Q, Chai Y, Hsu YT (2005). Distinct domains of Bcl-X_L_ are involved in Bax and Bad antagonism and in apoptosis inhibition.. Exp Cell Res.

[14] Pastorino JG, Shulga N, Hoek JB (2002). Mitochondrial binding of hexokinase II inhibits Bax-induced cytochrome c release and apoptosis.. J Biol Chem.

[15] Bernardi P, Krauskopf A, Basso E, Petronilli V, Blachly-Dyson E (2006). The mitochondrial permeability transition from *in vitro* artifact to disease target.. Febs J.

[16] Rasola A, Bernardi P (2007). The mitochondrial permeability transition pore and its involvement in cell death and in disease pathogenesis.. Apoptosis.

[17] Machida K, Ohta Y, Osada H (2006). Suppression of apoptosis by cyclophilin D via stabilization of hexokinase II mitochondrial binding in cancer cells.. J Biol Chem.

[18] Baines CP, Kaiser RA, Purcell NH, Blair NS, Osinska H (2005). Loss of cyclophilin D reveals a critical role for mitochondrial permeability transition in cell death.. Nature.

[19] Basso E, Fante L, Fowlkes J, Petronilli V, Forte MA (2005). Properties of the permeability transition pore in mitochondria devoid of cyclophilin D.. J Biol Chem.

[20] Nakagawa T, Shimizu S, Watanabe T, Yamaguchi O, Otsu K (2005). Cyclophilin D-dependent mitochondrial permeability transition regulates some necrotic but not apoptotic cell death.. Nature.

[21] Schinzel AC, Takeuchi O, Huang Z, Fisher JK, Zhou Z (2005). Cyclophilin D is a component of mitochondrial permeability transition and mediates neuronal cell death after focal cerebral ischemia.. Proc Natl Acad Sci U S A.

[22] Kokoszka JE, Waymire KG, Levy SE, Sligh JE, Cai J (2004). The ADP/ATP translocator is not essential for the mitochondrial permeability transition pore.. Nature.

[23] Hansson MJ, Mattiasson G, Mansson R, Karlsson J, Keep MF (2004). The nonimmunosuppressive cyclosporin analogs NIM811 and UNIL025 display nanomolar potencies on permeability transition in brain-derived mitochondria.. J Bioenerg Biomembr.

[24] Angelin A, Tiepolo T, Sabatelli P, Grumati P, Bergamin N (2007). Mitochondrial dysfunction in the pathogenesis of Ullrich congenital muscular dystrophy and prospective therapy with cyclosporins.. Proc Natl Acad Sci U S A.

[25] Azoulay-Zohar H, Israelson A, Abu-Hamad S, Shoshan-Barmatz V (2004). In self-defence: hexokinase promotes voltage-dependent anion channel closure and prevents mitochondria-mediated apoptotic cell death.. Biochem J.

[26] Robey RB, Hay N (2006). Mitochondrial hexokinases, novel mediators of the antiapoptotic effects of growth factors and Akt.. Oncogene.

[27] Juhaszova M, Wang S, Zorov B, Nuss H, Gleichmann M (2008). The identity and regulation of the mitochondrial permeability transition pore: Where the known meets the unknown.. Ann N Y Acad Sci. (in press).

[28] Postic C, Leturque A, Printz RL, Maulard P, Loizeau M (1994). Development and regulation of glucose transporter and hexokinase expression in rat.. Am J Physiol.

[29] Zorov DB, Filburn CR, Klotz LO, Zweier JL, Sollott SJ (2000). Reactive oxygen species (ROS)-induced ROS release: a new phenomenon accompanying induction of the mitochondrial permeability transition in cardiac myocytes.. J Exp Med.

[30] Halestrap AP, Kerr PM, Javadov S, Woodfield KY (1998). Elucidating the molecular mechanism of the permeability transition pore and its role in reperfusion injury of the heart.. Biochim Biophys Acta.

[31] Belzacq AS, Vieira HL, Kroemer G, Brenner C (2002). The adenine nucleotide translocator in apoptosis.. Biochimie.

[32] Baines CP, Kaiser RA, Sheiko T, Craigen WJ, Molkentin JD (2007). Voltage-dependent anion channels are dispensable for mitochondrial-dependent cell death.. Nat Cell Biol.

[33] Blachly-Dyson E, Zambronicz EB, Yu WH, Adams V, McCabe ER (1993). Cloning and functional expression in yeast of two human isoforms of the outer mitochondrial membrane channel, the voltage-dependent anion channel.. J Biol Chem.

[34] Orrenius S, Zhivotovsky B, Nicotera P (2003). Regulation of cell death: the calcium-apoptosis link.. Nat Rev Mol Cell Biol.

[35] Scorrano L, Ashiya M, Buttle K, Weiler S, Oakes SA (2002). A distinct pathway remodels mitochondrial cristae and mobilizes cytochrome c during apoptosis.. Dev Cell.

[36] Krauskopf A, Eriksson O, Craigen WJ, Forte MA, Bernardi P (2006). Properties of the permeability transition in VDAC1^-/-^ mitochondria.. Biochim Biophys Acta.

[37] Wu S, Sampson MJ, Decker WK, Craigen WJ (1999). Each mammalian mitochondrial outer membrane porin protein is dispensable: effects on cellular respiration.. Biochim Biophys Acta.

[38] Gramaglia D, Gentile A, Battaglia M, Ranzato L, Petronilli V (2004). Apoptosis to necrosis switching downstream of apoptosome formation requires inhibition of both glycolysis and oxidative phosphorylation in a BCL-X_L_- and PKB/AKT-independent fashion.. Cell Death Differ.

[39] Rasola A, Geuna M (2001). A flow cytometry assay simultaneously detects independent apoptotic parameters.. Cytometry.

[40] Fontaine E, Eriksson O, Ichas F, Bernardi P (1998). Regulation of the permeability transition pore in skeletal muscle mitochondria. Modulation By electron flow through the respiratory chain complex I.. J Biol Chem.

[41] Fields GB, Noble RL (1990). Solid phase peptide synthesis utilizing 9-fluorenylmethoxycarbonyl amino acids.. Int J Pept Protein Res.

[42] Petronilli V, Penzo D, Scorrano L, Bernardi P, Di Lisa F (2001). The mitochondrial permeability transition, release of cytochrome *c* and cell death. Correlation with the duration of pore openings *in situ*.. J Biol Chem.

